# Dynamic navigation versus conventional 3D-printed guides in implant surgery: A randomised controlled trial

**DOI:** 10.6026/973206300214333

**Published:** 2025-12-15

**Authors:** Aumkar Trivedi, Kondaveeti Viswaja, Nidhi Mangtani Kalra, Nasim Nasim, Abdul Kalam Azad, Gulshan Kumar Tomar, Vardharajula Venkat Ramaiah, Awadhesh Kumar Gupta

**Affiliations:** 1 Department of Dentistry, GMERS Medical College and Hospital, Vadnagar, India; 2 Department of General Pathology, SRM Dental College, Ramapuram, Chennai-600089, Tamilnadu, India; 3 Department of Prosthodontics, MMCDSR, Mullana, India; 4 Department of Oral and Maxillofacial Surgery, Inderprastha Dental College and Hospital, Ghaziabad, India; 5 Department of Oral and Maxillofacial Surgery, College of Dentistry, Qassim University, Saudi Arabia; 6 Department of Prosthodontics, Uttranchal Dental and Medical research Institute, Dehradun, Uttarakhand, India; 7 Department of Public Health, College of Applied Medical Sciences, Qassim University, Buraydah 51542, PO Box 6666, Saudi Arabia; 8 Department of Oral Pathology and Microbiology, DJ Dental College and Research, Modinagar, Ghaziabad, Utter Pradesh, India

**Keywords:** Dental implant, dynamic navigation, 3D-print, computer-assisted implantology, CBCT, patient satisfaction

## Abstract

Accurate implant positioning is essential for ensuring long-term prosthetic success and peri-implant health. Therefore, it is of interest to compare the precision, surgical efficiency and short-term clinical outcomes between dynamic navigation (DN) and static 3D-printed guides (SG) in single-tooth implant placement. Fifty patients were randomly assigned to either the DN (n=25) or SG (n=25) group and parameters such as coronal, apical and angular deviations, surgical time, implant stability (ISQ) and patient satisfaction were assessed. DN demonstrated significantly higher accuracy (coronal deviation 0.90±0.30 mm vs 1.40±0.40 mm; p<0.001) and reduced surgical time (18±4 min vs 22±5 min; p=0.002) compared with SG, while early implant stability and complication rates were similar between groups. Thus, we show that dynamic navigation enhances implant placement accuracy and efficiency without compromising short-term clinical outcomes, supporting its clinical advantage over conventional static guides.

## Background:

The accuracy of dental implant placement plays a pivotal role in determining long-term prosthetic success, esthetic outcomes and peri-implant health. With the increasing integration of digital technologies in dentistry, computer-assisted implant surgery (CAIS) has evolved as a transformative advancement, offering superior precision and predictability compared to traditional freehand approaches [1]. The two principal modalities of CAIS-static surgical guides (SG) and dynamic navigation (DN) systems-allow clinicians to execute preoperative implant planning with enhanced accuracy and reduced surgical error [2, 3]. Dynamic navigation systems enable real-time feedback and intraoperative adjustments, while static guides rely on pre-fabricated templates for transferring planned implant positions to the surgical field. Comparative studies have shown that both methods significantly improve implant placement accuracy; however, the degree of precision and clinical applicability remains a topic of ongoing investigation [1]. Younis *et al.* [1] demonstrated in a prospective clinical study that DN provides superior angular and linear accuracy compared to static surgical guides and freehand methods. Similarly, Khaohoen *et al.* [2], through a recent meta-analysis, confirmed that computer-aided static, dynamic and robot-assisted implant surgeries significantly outperform conventional techniques in placement accuracy, with DN offering greater flexibility and intraoperative control. Wu *et al.* [3] further validated that DN ensures better reproducibility and safety by providing continuous visual guidance, thereby minimizing deviations during osteotomy and implant insertion. In a systematic review and meta-analysis, Lau *et al.* [4] emphasized that both static and dynamic CAIS demonstrate high accuracy in immediate implant placement, though operator experience and case complexity influence precision. Similarly, Vinnakota *et al.* [5] reported that DN exhibits slightly higher positional and angular accuracy than SG, but variability in study designs and measurement parameters limits the strength of these conclusions. Kulkarni *et al.* [6] also observed consistent accuracy benefits with DN and SG compared to freehand surgery, though they highlighted methodological heterogeneity across studies, indicating the need for standardized clinical protocols. Furthermore, Yang and Geng [7] conducted a randomized controlled clinical trial in the posterior maxillary region and found that DN not only improved accuracy but also reduced surgical duration and intraoperative discomfort, highlighting its clinical efficiency and ergonomic advantage.

Despite these advancements, existing literature still presents limited clinical data directly comparing DN and SG under uniform clinical conditions, especially concerning operative metrics, prosthetic alignment and early clinical outcomes. Although recent systematic reviews and clinical studies [1-7] have consistently demonstrated that both static and dynamic navigation systems enhance implant placement accuracy compared to freehand surgery, there remains a notable research gap. Most studies have either focused on *in vitro* or pilot clinical evaluations with limited sample sizes, non-standardized measurement methods, or narrow outcome parameters confined to angular or coronal deviations. Therefore, it is of interest to compare surgical accuracy, operative efficiency and short-term clinical outcomes between dynamic navigation and static surgical guide-assisted implant placement in a standardized clinical environment.

## Materials and Methods:

Prospective parallel RCT. Institutional ethics committee approval obtained prior to the study. Informed consent was obtained from all participants. Inclusion criteria were; Adult patients (≥18 years) requiring single-tooth implant in healed sites (≥3 months), adequate bone volume for implant placement without major augmentation and ability to attend follow-up visits. Exclusion criteria were; active infection at site, immediate extraction/implantation cases, Systemic contraindications to implant surgery and severe parafunctional habits or uncontrolled diabetes. Sample size (n=50; 25 per arm) chosen to detect a clinically meaningful difference of 0.4 mm in coronal deviation with 80% power and α=0.05 (assumptions based on prior literature and pilot data). Random sequence created by computer; sealed envelopes used for allocation concealment. All patients had a preoperative CBCT and intraoral scan. Implant planning (position, angulation and depth) was performed in the planning software. For the SG group, patient-specific tooth-supported or mucosa-supported surgical guides were designed and 3D-printed; guided sleeves were used per manufacturer protocol. For the DN group, the plan was exported to the dynamic navigation system and fiducial registration performed intraoperatively. All surgeries were performed by experienced implant surgeons trained in both systems. Local anesthesia with standard sterile technique used. Implants of similar dimensions and from the same implant system were used across groups.

## SG arm:

Guide fit checked, osteotomy performed through guide sleeves according to drill sequence; implant placed through guide.

## DN arm:

Registration performed using fiducial markers or registration splints; tracking calibration performed; osteotomy and implant placement executed under real-time navigation guidance.

## Outcome measurements:

[1] Coronal (platform) linear deviation (mm)

[2] Apical linear deviation (mm)

[3] Angular deviation (degrees)

## Secondary outcomes:

[1] Surgical time (skin-to-skin, minutes)

[2] Intraoperative and early postoperative complications (nerve injury, bleeding, guide misfit, implant malposition requiring correction)

[3] Implant primary stability (ISQ) measured at placement

[4] Patient satisfaction (10-point Likert at 1-week)

Continuous variables reported as mean ± standard deviation. Between-group comparisons used independent sample t-tests (or Mann-Whitney U if non-normal). Categorical variables used chi-square or Fisher exact test. A p-value <0.05 was considered statistically significant. Analysis performed using standard statistical software.

## Results:

Fifty patients were randomised and completed the primary outcome assessment (no losses to follow-up in the immediate postoperative phase). Demographics were balanced between groups. Both groups were comparable at baseline with respect to age, gender distribution and implant site location. No statistically significant differences were observed between the Dynamic Navigation and 3D Printed Guide groups (p > 0.05), confirming homogeneity of the sample before surgical intervention (Table 1 - see PDF). Dynamic navigation demonstrated significantly lower coronal, apical and angular deviations compared to the 3D printed guide group (p < 0.001 for all). The mean difference favored dynamic navigation by approximately 0.5-0.6 mm in linear accuracy and 1.7° in angular precision. These findings indicate superior spatial accuracy and alignment control with real-time navigation (Table 2 - see PDF). Dynamic navigation significantly reduced mean surgical time compared with the 3D printed guide group (18 ± 4 min vs. 22 ± 5 min, p = 0.002). Minor intraoperative guide fit errors and postoperative complications were infrequent in both groups and did not differ significantly (p > 0.05). Overall, both techniques demonstrated high procedural safety, with dynamic navigation offering greater intraoperative efficiency (Table 3 - see PDF). Both groups achieved comparable immediate implant stability, with no significant difference in mean ISQ values (p = 0.25). However, patient-reported satisfaction scores at one week were significantly higher in the dynamic navigation group (p = 0.01), reflecting better perceived comfort and postoperative experience (Table 4 - see PDF). Data were assessed for normality using the Shapiro-Wilk test; primary outcomes were approximately normally distributed. Independent t-tests were used for between-group comparisons of continuous variables; unequal variances were checked with Levene's test. Categorical comparisons used chi-square; if expected counts <5, Fisher's exact test was applied. Confidence intervals (95%) for mean differences estimated using pooled standard errors.

## Discussion:

Precision in dental implant placement is crucial for ensuring optimal prosthetic fit, long-term osseointegration and peri-implant health. Computer-assisted technologies, including static 3D-printed surgical guides (SG) and dynamic navigation (DN), have been introduced to enhance placement accuracy while minimizing surgical invasiveness. Static guides, fabricated from CBCT and intraoral scan data, transfer pre-planned implant positions with high reliability but are limited by intraoperative rigidity and potential fit discrepancies. In contrast, DN systems provide real-time visual tracking of drills and implants relative to the patient's anatomy, allowing intraoperative adjustments that may overcome static guide limitations [1- 3]. This randomized controlled trial compared these two digital modalities across 50 single-implant cases, assessing deviations, operative parameters and early clinical outcomes. As shown in Table 1 (see PDF), both groups were statistically comparable in baseline demographics, implant site distribution and gender composition, confirming appropriate randomization and balanced study arms. Dynamic navigation achieved significantly lower coronal, apical and angular deviations than the 3D-printed guide (Table 2 - see PDF). This underscores the enhanced spatial control achievable through real-time navigation. Comparable findings were reported by Younis *et al.* [1], who observed greater accuracy in DN compared with static systems and by Khaohoen *et al.* [2], who demonstrated superior precision with navigation and robotic systems over static and freehand methods. Similarly, Wu *et al.* [3] emphasized the angular accuracy benefits of DN in partially edentulous cases.Our results-mean coronal deviation 0.90 mm vs 1.40 mm, apical 1.10 mm vs 1.70 mm and angular 2.8° vs 4.5°-mirror those of recent meta-analyses by Lau *et al.* [4] and Kulkarni *et al.* [6], who reported pooled deviations below 1 mm and 3° for DN, representing clinically meaningful accuracy improvements. Dynamic navigation reduced mean surgical time (18 ± 4 min vs 22 ± 5 min, p = 0.002), suggesting a more efficient intraoperative workflow (Table 3 - see PDF). The absence of guide-fit errors in DN contrasts with occasional template misfits in SG, a limitation frequently discussed by Yang and Geng [7]. Static templates, though reliable, require flawless pre-surgical fit verification and can restrict intraoperative flexibility. In this study, DN's adaptability provided smoother adjustments during osteotomy and implant placement, enhancing ergonomic control without increasing complications. Early postoperative outcomes were favorable in both groups, with low complication rates (Table 3 - see PDF). Immediate implant stability (ISQ) values were comparable (72 ± 6 vs 70 ± 7, p = 0.25), consistent with findings by Banerjee *et al.* [8], who noted that navigation precision does not necessarily alter primary stability. However, patient satisfaction scores were significantly higher in the DN group (9.1 ± 0.7 vs 8.5 ± 0.9, p = 0.01; Table 4 - see PDF), reflecting patient preference for shorter, minimally invasive and technologically guided procedures-echoing trends reported by Deng *et al.* [9] and Khan *et al.* [10].

Overall, our findings strengthen the existing body of evidence that DN improves surgical precision and procedural efficiency while maintaining equivalent clinical safety and early stability. Previous comparative reviews by Kulkarni *et al.* [6] and Kivovics *et al.* [11] concluded that both DN and SG yield clinically acceptable accuracy, though DN offers the advantage of intraoperative flexibility and error correction. Moreover, as operator experience and bone density significantly influence static guide precision [12], DN provides a technological solution to partially mitigate these operator-dependent variabilities. These results also complement systematic analyses highlighting that navigation technology offers clinically meaningful accuracy within prosthetic tolerance limits, often below 1 mm, thereby reducing potential restorative discrepancies [13, 14]. From a clinical perspective, DN demonstrates clear advantages for single-tooth and esthetic zone placements where spatial precision is paramount. Although static guides remain cost-effective for straightforward cases, DN may justify its investment through improved predictability and workflow adaptability. Integration with augmented reality and AI-assisted navigation, as proposed in current technological trends, could further refine precision and user experience. All continuous variables passed the Shapiro-Wilk test for normality. Independent t-tests compared mean values and categorical variables were assessed using chi-square or Fisher's exact tests. Significant intergroup differences were found for all primary accuracy parameters (p< 0.001), with non-significant differences for ISQ and complications. Further multicenter trials with larger cohorts and long-term follow-up are warranted to examine marginal bone loss, prosthesis longevity and cost-effectiveness. Standardization of measurement protocols and calibration across systems will also enhance reproducibility and meta-analytic comparability. Early implant primary stability (ISQ) was comparable between groups, suggesting that improved accuracy did not necessarily translate to superior immediate mechanical stability in healed sites when implants of similar dimensions were used. Patient satisfaction favored DN, possibly because of perceived shorter procedure time and lower anxiety when real-time guidance is used. Jaiswal *et al.* stated that digital impression helps for better implant placemen compared to conventional method [15]. Dental implants are comolntused to replace missing teeth [16, 17, 18 and 19]. The success of implant prosthesis is depends on local factors such as implant design, implant type, bone quality, oral health, periodontal condition and systemic factors such as medical condition of patients [16, 17 and 20]. Operator quality also helps in implant success. 3D printed surgical guide implant design helps to deliver good quality implants. For long term success of implant, patient should maintain good oral health using mouth rinse [21, 22 and 23]. Complication rates were low in both groups; no major nerve injuries or vital structure violations occurred. Two guide cases required minor intraoperative adjustment due to slight guide seating mismatch - a known limitation of static guides dependent on fit accuracy. The study limitations are; smaller sample size, while adequate for selected accuracy endpoints, is modest and from a single center; multi-center validation would strengthen generalizability. Only healed single-tooth cases were included; immediate placement, complex augmentation and edentulous arches were excluded. Follow-up in this report focuses on early outcomes - longer-term survival, marginal bone loss and prosthetic outcomes require extended follow-up. For clinicians balancing accuracy, flexibility and time, DN offers advantages in accuracy and potentially in workflow efficiency. Static guides remain a viable and cost-effective option, particularly when guide fit can be guaranteed and complex intraoperative adjustments are not anticipated. Choice should consider case complexity, cost, clinician familiarity and available infrastructure.

## Conclusion:

Dynamic navigation significantly improved positional and angular accuracy and reduced surgical time compared with conventional 3D-printed static guides in this randomized controlled trial of 50 implants. Techniques demonstrated high safety and comparable implant stability, yet dynamic navigation provided superior real-time control, intraoperative adaptability and patient satisfaction. Thus, we show dynamic navigation as a clinically advantageous digital approach for achieving precision and efficiency in routine implant surgery. Further multicenter studies with larger samples and long-term follow-up are recommended to validate these outcomes and assess the cost-effectiveness and long-term prosthetic success of navigation-assisted implantology.
